# Novel siRNA formulation to effectively knockdown mutant p53 in osteosarcoma

**DOI:** 10.1371/journal.pone.0179168

**Published:** 2017-06-21

**Authors:** Anup K. Kundu, Swathi V. Iyer, Sruti Chandra, Amit S. Adhikari, Tomoo Iwakuma, Tarun K. Mandal

**Affiliations:** 1 Center for Nanomedicine and Drug Delivery, Xavier University College of Pharmacy, New Orleans, Louisiana, United States of America; 2 Department of Biology, Xavier University of Louisiana, New Orleans, Louisiana, United States of America; 3 Department of Genetics and Stanley S. Scott Cancer Center, Louisiana State University Health Sciences Center, New Orleans, Louisiana, United States of America; Universidad de Castilla-La Mancha, SPAIN

## Abstract

**Objectives:**

The tumor suppressor p53 plays a crucial role in the development of osteosarcoma. The primary objective of this study is to develop and optimize lipid based nanoparticle formulations that can carry siRNA and effectively silence mutant p53 in 318–1, a murine osteosarcoma cell line.

**Methods:**

The nanoparticles were composed of a mixture of two lipids (cholesterol and DOTAP) and either PLGA or PLGA-PEG and prepared by using an EmulsiFlex-B3 high pressure homogenizer. A series of studies that include using different nanoparticles, different amount of siRNAs, cell numbers, incubation time, transfection media volume, and storage temperature was performed to optimize the gene silencing efficiency.

**Key findings:**

Replacement of lipids by PLGA or PLGA-PEG decreased the particle size and overall cytotoxicity. Among all lipid-polymer nanoformulations, nanoparticles with 10% PLGA showed highest mutant p53 knockdown efficiency while maintaining higher cell viability when a nanoparticle to siRNA ratio equal to 6.8:0.66 and 75 nM siRNA was used. With long term storage the mutant p53 knockdown efficiency decreased to a greater extent.

**Conclusions:**

This study warrants a future evaluation of this formulation for gene silencing efficiency of mutant p53 in tissue culture and animal models for the treatment of osteosarcoma.

## 1. Introduction

The tumor suppressor gene p53 is a key regulator of early stages of osteogenic differentiation and defends our body from the development of osteosarcoma. Mutations or deletion of p53 has been associated with the pathogenesis of numerous human cancers, including osteosarcomas[1]. Mutations in p53 lead to genomic instability[2] and stimulate unrestricted osteoblastic proliferation[3]. In the United States, approximately 400 new cases of osteosarcoma are registered per year[4]. Although mutations in p53 have been reported to be 20–50% in human osteosarcoma[5], a recent study has found that over 90% of osteosarcomas have either sequence mutations or structural variations (mainly in the first intron) in the p53 gene[6].

Osteosarcoma is treated with a combination of therapies that can include surgical excision, chemotherapy and radiation therapy. Tumors with p53 mutations show tendency to be resistance to chemotherapy and despite the available standard care high grade osteosarcoma rapidly disseminates leading to poor overall prognosis. New forms of therapies are sought to improve the treatment of osteosarcoma including angiogenesis inhibitors, drugs that act on bone microenvironment, receptor tyrosine kinase inhibitors, immune-system modulators, and various chemo-sensitizers[7]. In order to minimize systemic toxicity, the tumors need to be addressed locally. This gives scope for targeted drug delivery, and this is where gene therapy steps in.

Gene therapy has led to significant advances in the treatment of infectious disease[8] and cancer[9]. Gene therapy techniques aimed at the introduction of a wild-type p53 gene into cancer cells have been implemented in lung[10], breast[11], esophageal, colorectal and prostate cancer[12]. However, very few clinical trials of gene therapy for osteosarcoma have been reported[13].

Appropriate gene delivery methods are the key to success in gene therapy. A number of techniques for DNA delivery have been attempted, such as electroporation, viral genomes, ballistic gold particles, liposomal and polymeric nanoparticles, and even direct injection of naked DNA. Viral vectors have been observed to be highly efficient, but they are also associated with high toxicity[14] and immunogenicity[15]. These limitations of using viral vectors for effective DNA delivery led to the development of nonviral vectors, such as lipid nanoparticles[16], and polymeric delivery vehicles[17]. Lipid mediated delivery of DNA is faster than viral delivery[18], and liposomal delivery vehicles are also preferred for decades because of their safety, non-immunogenicity, comparatively easy assembly, and commercial large scale production capability[19].

The field of small interfering RNAs (siRNAs) which induce post-transcriptional gene silencing in a sequence specific manner is rapidly emerging. The mechanism of action of siRNA consists of an initial step in which double-stranded RNA (dsRNA) cleaved into 21 nt fragments of siRNA, followed by the incorporation of antisense strand or guide strand into RNA Induced Silencing Complex (RISC complex), the guiding sequence then recognizes and binds to homologous mRNA that is subsequently degraded[20].

Some challenges faced during clinical application of siRNAs include their low transfection efficiency, poor tissue penetration, and nonspecific immune stimulation. Their potential as anticancer therapeutics hinges on the availability of a carrier vehicle that can be systemically and safely administered in a repeated fashion to deliver siRNA specifically and efficiently to the tumor, both primary and metastatic ones. Although advances are being made, currently, only a few approaches have been potentially feasible in patients[21]. Cationic nanoparticles/cationic liposomes having high transfection efficiency into tumor cells[22] can form nanoplexes/lipoplexes with siRNA and have the potential of use as siRNA delivery vehicle. Lipid-polymer hybrid nanoparticles have been used to co-deliver siRNA and Gemcitabine for effective treatment of pancreatic cancer[23].

Naked siRNA is negatively charged which hinders its cellular internalization and therefore needs a protective carrier. Nanoparticles bearing a positive surface charge encapsulate siRNA by electrostatic interaction and are believed to facilitate uptake by negatively charged cell membranes[24]. Escaping the endogenous nuclease digestion is vital during delivery of siRNA into target cells or organs in order to maintain its functional integrity, and a protective carrier is also required to overcome this barrier. A delivery vehicle helps to prolong the serum and intracellular half-life of siRNA by improving pharmacokinetics and nuclease resistance making the RNAi effect last longer than naked siRNAs[25]. Lipid-encapsulated siRNAs have longer serum half-life (6.5h) than naked siRNAs (0.8h)[26]. Sustained release of siRNA prolonging the RNAi effect could be achieved with PLGA copolymer microspheres[27] possessing a matrix through which siRNA slowly diffuses.

Polymers are ideal as nucleic acid carriers because their units by unit construction offer the scope to fine-tune their properties for efficient transfection and release of siRNA. Clinically validated biodegradable and biocompatible materials like poly(lactic-co-glycolic acid) (PLGA) widely used in drug delivery and biomedical devices, approved by the US Food and Drug Administration (FDA)[28] would be perfectly suitable for siRNA delivery. PLGA spontaneously complex with nucleic acids and subsequently facilitate cellular uptake by negatively charged cell membranes[25]. Core-shell structured nanoparticles containing block co-polymers like poly(ethylene glycol) or PEG[29], forms a protective outer coating around the polyplex core containing polymers complexed with siRNA and shields it by steric-stabilization.

In the current study, we designed and selected a lipid-polymer hybrid nanoformulation from a series of various test nanoparticles capable of carrying siRNA to knockdown mutant p53 in a mouse osteosarcoma cell line. This nanocarrier was optimized so as to maintain small particle size, high siRNA encapsulation, and effective gene knockdown efficiency while minimizing cell cytotoxicity. Evaluation of the effect of media volume on the transfection efficiency of the nanoparticles, long term storage effects of nanoparticles on cell viability and knockdown efficiency were also undertaken by this study.

## 2. Materials and methods

### 2.1. Materials

The reagents for cell culture including fetal bovine serum albumin (FBS), Dulbecco’s modified Eagle’s medium (DMEM) and penicillin/streptomycin antibiotics were purchased from Gibco, Invitrogen Corp. (Carlsbad, CA, USA). The chemicals required for synthesis of nanoparticles including 1,2-dioleoyl-3-trimethylammonium-propane (DOTAP) and cholesterol were ordered from Avanti Polar-lipids Inc. (Birmingham, AL, USA). Protamine sulfate salt Grade X, trehalose dihydrate and HPLC grade chloroform were obtained from Sigma Chemical Co. (St. Louis, MO, USA). Poly (lactic-co-glycolic acid) or PLGA and poly (ethylene glycol) coated PLGA or PLGA-PEG were obtained from Boehringer Ingelheim (Germany). The murine *p53*-specific siRNA (sequence: *GUCUGUUAUGUGCACGUAC*) and control *non-target* siRNA were purchased from Dharmacon RNAi Technologies (Lafayette, CO). The Ribogreen assay kit was supplied by Molecular Probes (Eugene, OR, USA). The antibodies used were: p53 (CM5, Vector Biolabs, Burlingame, CA), β-actin (A2228, Sigma Aldrich, St. Louis, MO), Vinculin (10R-C105a, Fitzgerald, Acton, MA), and HRP goat anti-rabbit or anti-mouse IgG (Thermo Fisher Scientific, Waltham, MA). All other reagents were of analytical grade and were supplied by Sigma Chemical Co. (St. Louis, MO, USA).

### 2.2. Preparation of hybrid liposome

The control liposome (F1) was prepared at 20 mM concentration, by using DOTAP and cholesterol mixed in equimolar proportions (Table 1). The other lipid-polymer hybrid liposomes were derived from F1 after gradually decreasing the amount of DOTAP and cholesterol and proportionately replacing with either PLGA as in F3, F4, F5, or PLGA-PEG as in F6, F7 and F8. EmulsiFlex-B3 high pressure homogenizer was used for preparing the liposomes. DOTAP, cholesterol and PLGA or PLGA-PEG were weighed into round bottom flask, dissolved in 15 ml HPLC grade chloroform, dried under nitrogen and then subjected to overnight vacuum. The lipid-polymer films were hydrated in DEPC treated water. The lipid polymer mixer was warmed and mixed at 50°C for 45 minutes by rotation and then kept at RT for 2 hours. The resultant dispersion was transferred into a scintillation vial and warmed again at 50°C for 15 minutes. The final lipid polymer dispersion was homogenized by using a high pressure homogenizer at 20,000 PSI for 5 cycles. Each time, 2.5 ml of lipid polymer dispersion was subjected to homogenization and the resultant hybrid liposomes were collected in another scintillation vial. They were kept at room temperature for 1 hour prior to overnight storage at 4°C. Next day, the liposomes were warmed at RT for 2 hours followed by adding trehalose solution prepared in DEPC-treated water. The liposomes were mixed with trehalose by vortexing and then kept in -80°C for 2 hours followed by lyophilization (Labconco freeze dryer; Labconco Corp., Kansas City, MO) for 5 days. After lyophilization, the dry particles were mixed thoroughly by using a sterilized spatula and stored in a desiccator at 4°C.

**Table 1:** Composition of different hybrid nanoparticles.

Blank nanoparticles	DOTAP (mg)	Cholesterol (mg)	PLGA (mg)		PLGA-PEG (10% diblock) (mg)	Nanoparticles volume in water (ml)	Trehalose (g)
F1	100	55.36				10	1.16
F3	95	52.59	5			10	1.16
F4	90	49.82	10			10	1.16
F5	80	44.288	20			10	1.16
F6	95	52.59		5		10	1.16
F7	90	49.82		10		10	1.16
F8	80	44.288		20		10	1.16

### 2.3. Preparation of siRNA entrapped nanoparticles

The composition of siRNA-entrapped nanoparticles was shown on Table 2. siRNA was condensed with freshly prepared protamine sulphate (PS) by dropwise addition of PS to siRNA in water. The mixture was vortexed at moderate speed and incubated for 40 min at room temperature (RT). The lyophilized hybrid liposomes were reconstituted in DEPC-treated water and kept at RT for 1 hour. The hybrid liposomes were sonicated in ice-cold water briefly for 1 min and added to the siRNA-PS complex followed by pipetting 30 times. Then the siRNA loaded nanoparticles were vortexed four to five times for thorough mixing. Finally, they were sonicated in ice cold water for 3 to 4 min to reduce particle size and kept on ice until their use for experiments.

**Table 2:** The toxicity and p53 knockdown efficiency of different hybrid nanoparticles.

siRNA encapsulated nanoparticles	Blank nanoparticles	siRNA conc. (nM)	% of Cell viability (Control siRNA encapsulated)	% of cell viability (p53-specific siRNA encapsulated)	Knockdown efficiency (%)
T1-1	F1 (6.8 μg)	100	85±4	88±3	40±4
T1-2	F1 (6.8 μg)	125	78±3	76±5	48±5
T1-3	F1 (6.8 μg)	150	76±4	77±6	63±3
T2-1	F1 (8.6 μg)	100	85±6	86±5	50±6
T2-2	F1 (8.6 μg)	125	75±4	78±3	59±5
T2-3	F1 (8.6 μg)	150	78±3	78±5	70±6
T3	F3 (6.8 μg)	150	72±5	70±4	65±4
T4	F4 (6.8 μg)	150	87±5	88±4	75±5
T5	F5 (6.8 μg)	150	83±6	85±5	60±4
T6	F6 (6.8 μg)	150	71±3	70±6	80±3
T7	F7 (6.8 μg)	150	84±3	82±4	76±6
T8	F8 (6.8 μg)	150	83±5	85±8	72±5

P.S. formulation also contains protamine sulphate (2 μg) each

### 2.4. Measurement of siRNA quantity in different hybrid nanoparticles

**Fig 1:**
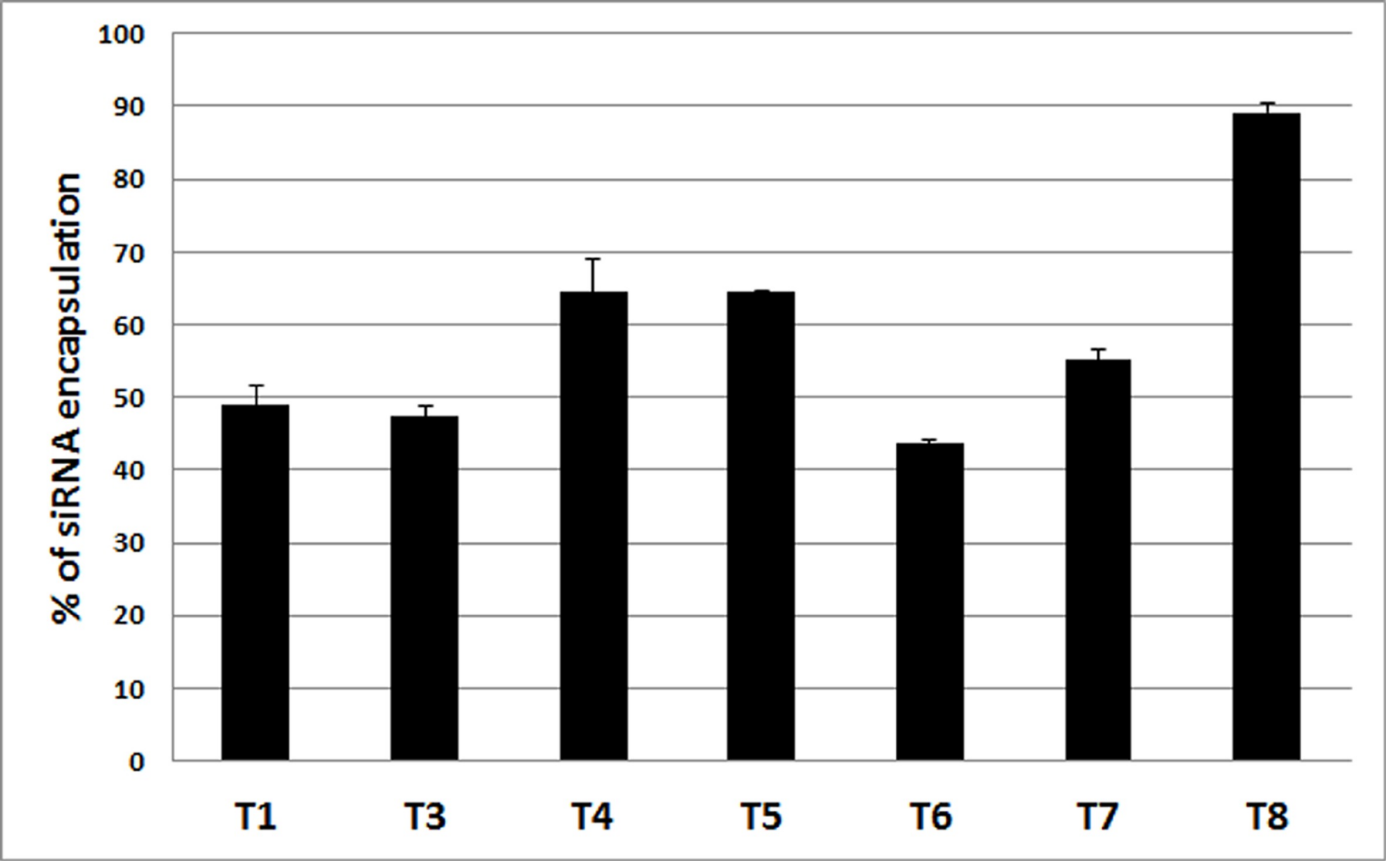
Determination of siRNA’s encapsulation efficiency of different hybrid nanoparticles. The encapsulated siRNA in different formulations (T1-T8) was decomplexed by exposure to 1% SDS for 18 h and then measured by Ribogreen Assay. The results represent mean ± standard deviation (n = 4).

The percent of siRNA encapsulated into the nanoparticles and that remaining in solution was measured. The efficiency of entrapment was calculated by comparing the amount of siRNA originally added to the sample and the amount of siRNA actually present in the nanoparticles. Briefly, after preparation, the samples (T1-T8) were centrifuged at 14,000 rpm (Allegra Centrifuge, Beckman Coulter Inc., Fullerton, CA) for 15 minutes at 4°C. Supernatants containing the free siRNA were separated from the pellets. 500 μl of a 1% sodium dodecyl sulfate (SDS) solution was added to the pellets, and to the supernatants. Samples were then incubated at 37°C for 18 hours with gentle agitation (50 rpm). The siRNA amount from both supernatants and pellets was measured by using Ribogreen assay following the manufacturer’s protocol. The results are reported (Fig 1) as the mean ± standard deviation (n = 4).

### 2.5. Determination of particle size, morphology and zeta potential

The particle size of the hybrid liposomes reported as mean ± standard deviation (n = 4), before and after siRNA loading (Table 3) was analyzed by dynamic laser light scatter at RT using a Delsa Nano C Particle Analyzer (Beckman Coulter Inc., Fullerton, CA, USA).

**Table 3:** Comparison of particle size between blank nanoparticles and siRNA-loaded nanoparticles.

		30% Size (nm)		70% Size (nm)		90% Size (nm)	
Blank nanoparticles	siRNA- loaded nanoparticles	Blank	siRNA-loaded	Blank	siRNA-loaded	Blank	siRNA-loaded
F1	T1	69±1	50±11	123±8	108±28	173±5	240±84
F3	T3	66±6	52±2	146±15	101±13	256±35	162±35
F4	T4	80±3	58±1	182±14	100±8	323±43	155±31
F5	T5	72±4	60±1	164±19	125±11	328±54	210±32
F6	T6	90±5	79±2	207±12	213±14	374±30	443±21
F7	T7	88±5	89±2	195±28	217±4	364±78	442±14
F8	T8	103±12	84±3	216±13	216±8	360±41	426±33

**Fig 2:**
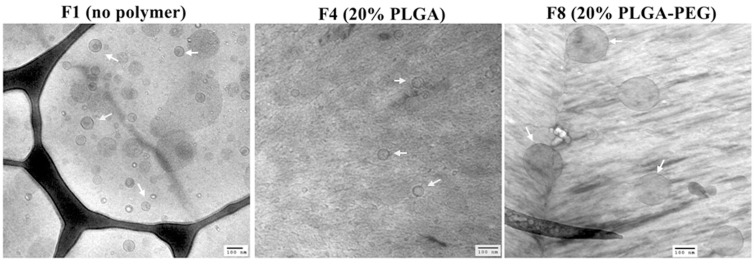
Cryo-TEM pictures of different blank hybrid nanoparticles. Scale bar represents 100 nm.

The morphology of three hybrid liposomes F1 (no polymer), F5 (20% PLGA) and F8 (20% PLGA-PEG) was examined by using Transmission Electron Microscope (JEOL 2010, Gatan) (Fig 2). The nanoparticles were reconstituted in water and a 6 μl drop of the formulation was placed on a holey carbon grid and rapidly vitrified in liquid ethane. The sample was then transferred under liquid nitrogen to the cryo-TEM sample holder and inserted into the cryo-TEM. The temperature of the sample grids was maintained at -175°C during the course of imaging.

For measuring the surface charge density of the nanoparticles (Table 4) before and after siRNA encapsulation using a Delsa Nano C Particle Analyzer (Beckman Coulter Inc., Fullerton, CA, USA), the system was precalibrated with standards. Then the zeta potential of all the samples was measured 5 times in 1mM KCl.

**Table 4:** Comparison of zeta potential between blank nanoparticles and siRNA-loaded nanoparticles.

Blank nanoparticles	siRNA-loaded nanoparticles	Zeta potentials (mV) Blank nanoparticles	Zeta potentials (mV) siRNA-loaded nanoparticles
F1	T1	56.6±3.6	41.6±3.2
F3	T3	62.6±5.2	39.9±2.6
F4	T4	68.7±4.5	45.8±1.8
F5	T5	63.5±5.6	46.4±2.3
F6	T6	42.1±3.0	43.1±3.7
F7	T7	43.7±4.5	44.1±3.5
F8	T8	42.3±5.0	43.3±3.0

### 2.6. Cell culture

The mouse osteosarcoma cell line 318–1 that carries p53R172H mutant alone[30, 31] was maintained in DMEM supplemented with 10% fetal bovine serum (FBS) and 1% penicillin-streptomycin (10,000 units/mL) in a humidified incubator at 37°C and 5% CO_2_ in 35 mm culture plates.

### 2.7. Measurement of cell viability

The effect of the nanoformulations on cell viability was assessed in a mouse osteosarcoma cell line 318–1[30]. 318–1 cells (1.5x10^5^, or different cell numbers), seeded on 6-well plates were transfected with either control non-target siRNA or mouse *p53*-specific siRNA[30] using hybrid nanoparticles. Forty-eight hours after transfection, live cell numbers were counted following Trypan blue staining using a hemocytometer counting entire field. The percentages of Trypan Blue negative cells in total cell number were calculated and standardized by that of non-treated cells.

### 2.8. Western blot analysis

Cell lysis was performed using radioimmunoprecipitation assay (RIPA) buffer, which consists of phosphatase and protease inhibitors (EMD Chemicals, San Diego, CA). 20–100 μg of protein from the cell lysate was separated by electrophoresis following loading onto 12% tris-glycine gel (Bio-Rad Laboratories, Inc, Hercules, CA), and transferred to polyvinylidene fluoride (PVDF) membrane (GE Healthcare Life Sciences). After blocking in 5% non-fat milk in 1×Tris-buffered saline (TBS) with 0.1% Tween-20 (TBS-T), the membrane was then blotted with antibodies for p53 (CM5, 1:5,000), β-actin (A2228, Sigma Aldrich, dilution 1:1,000) or Vinculin (10R-C105a, 1:2,000) followed by appropriate horseradish peroxidase (HRP) conjugated goat anti-rabbit (1:5,000) or anti-mouse IgG (1:10,000). Blots were washed in TBS-T, following which they were imaged using Gel Doc™ XR+ System (Bio-Rad Laboratories, Inc, Hercules, CA).

## 3. Results

**Fig 3:**
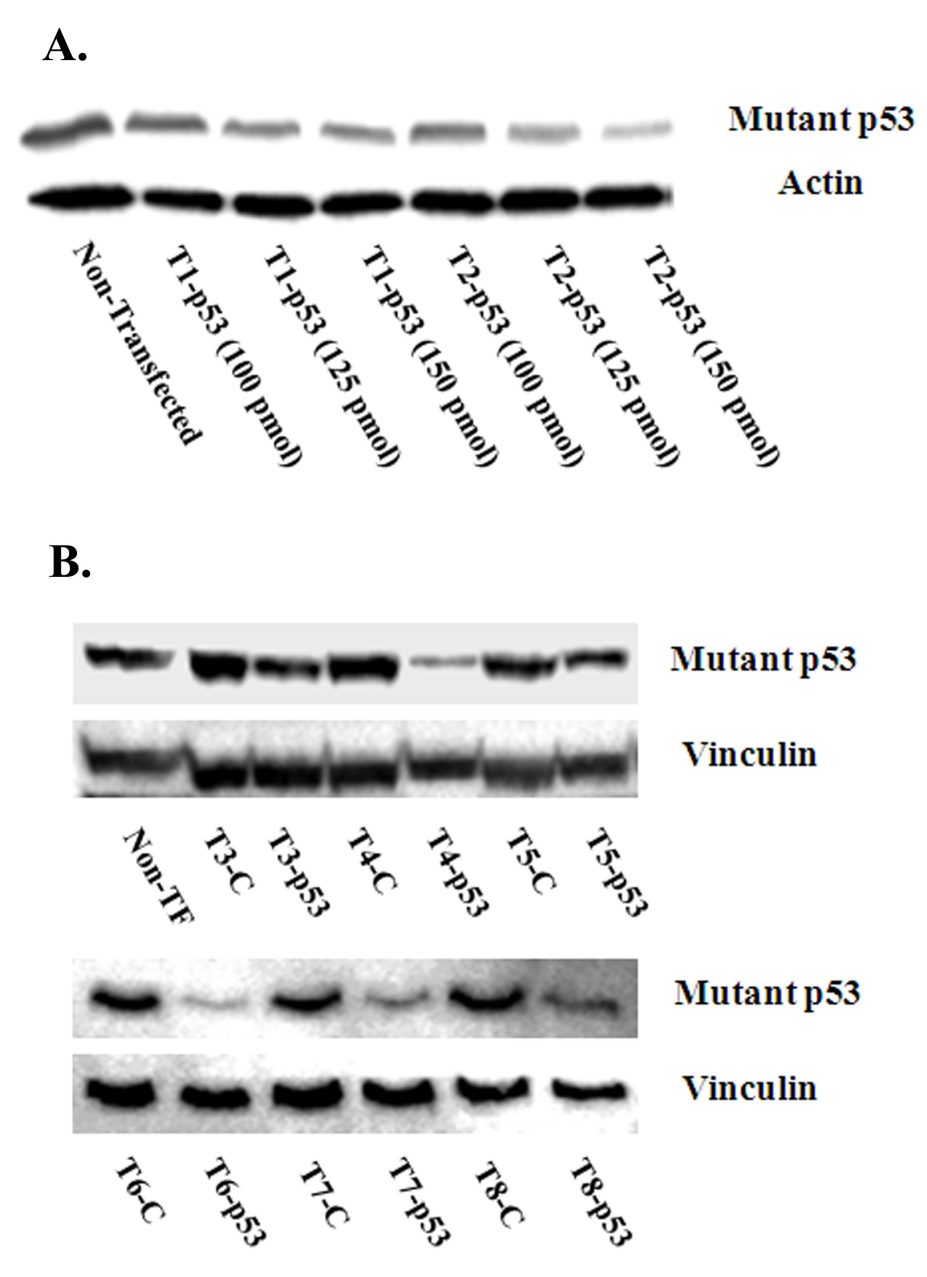
Identification of the formulation with the best knockdown efficiency by western blotting following nanoparticle transfection with different formulation (A. T1 and T2; B. T3-T8). 318–1 cells were transfected with different formulations encapsulating either three different concentrations of p53 siRNA (*i*.*e*. 100, 125 and 150 pmol) (Fig 3A) or 150 pmol siRNA (Fig 3B). Representative western blotting results for p53, actin and vinculin are shown. This experiment was performed 3 independent times.

In this study, we have tried to substitute lipid in our original lipid based nanoformulations with polymer to improve the delivery efficiency as well as the cell viability of the transfected cells[32]. Nontoxic PLGA (F3-F5) and PLGA-PEG (F6-F8) were proportionately added (Table 1) to reduce the lipid content of the mother particle F1. Trial nanoparticles were initially prepared incorporating 6.8 μg F1 (three T1 formulations) and 8.6 μg F1 (three T2 formulations) with varying concentrations of siRNA targeting mouse p53 in 318–1 cells carrying mutant p53 (p53R172H) (Table 2). All the formulations of T1 and T2 showed good knockdown efficiency and mutant p53 silencing efficiency with the highest knockdown achievable at 150 nM siRNA (Fig 3A).

### 3.1. Cell viability and knockdown efficiency of different nanoparticles

The knockdown efficiency of different hybrid nanoparticles and subsequent cell viability was measured in 318–1 cells (Table 2). At 6.8 μg lipid, the siRNA concentration was increased from 100 to 150 nM (T1-1 to T1-3), and the best knockdown of mutant p53 (~63±3%) was attained by T1-3 with cell viability ~77±6% (Table 2 and Fig 3A). Likewise, of the three combinations tested with T2 (lipid amount 8.6 μg), T2-3 encapsulating 150 nM siRNA showed the highest knockdown (~70±6%) with a cell viability ~78±5% (Table 2 and Fig 3A). In both T1 and T2 formulations, there was, however, a decrease in viable cells with increasing siRNA concentration from 100 to 150 nM. Since 150 nM siRNA gave the highest inhibition of mutant p53, further experiments on cell viability and knockdown efficiency using hybrid nanoparticles have been done with 150 nM siRNA. However, T1-3 (6.8 μg lipid) was chosen over T2-3 (8.6 μg lipid) to prepare lipid-polymer hybrid nanoparticles because of its reduced lipid content (Table 2).

Polymer substitution of the nanoparticles improved the cell viability as well as the knockdown efficiency. T3, T4 and T5 with 5%, 10% and 20% PLGA substitution showed cell viability of ~70±4%, ~88±4% and ~85±5% and knockdown efficiency ~65±4%, ~75±5% and ~60±4%, respectively (Table 2 and Fig 3B). Again, T6, T7 and T8 with 5%, 10% and 20% PLGA-PEG registered cell viability ~70±6%, ~82±4% and ~85±8% and knockdown efficiency ~80±3%, ~76±6% and ~72±5%, respectively (Table 2 and Fig 3B). Both T4 (within the PLGA group) and T6 (within the PLGA-PEG group) showed the highest knockdown efficiency to silence mutant p53, however, T4 (10% PLGA) between T4 and T6 stands out as the nanoparticle with the least cytotoxicity and best suited for siRNA delivery into the osteosarcoma cell line. The formulations with p53-targeted siRNA were compared with another set of formulations having non-targeted (p53) siRNA to see whether the p53 specific siRNA itself could create toxicity to the cells (Table 2). However, there were no differences observed in the cell viability whether or not the formulations were prepared with p53 specific or non-specific siRNA (Table 2).

### 3.2. Silencing of mutant p53 by using different siRNA concentration

**Fig 4:**
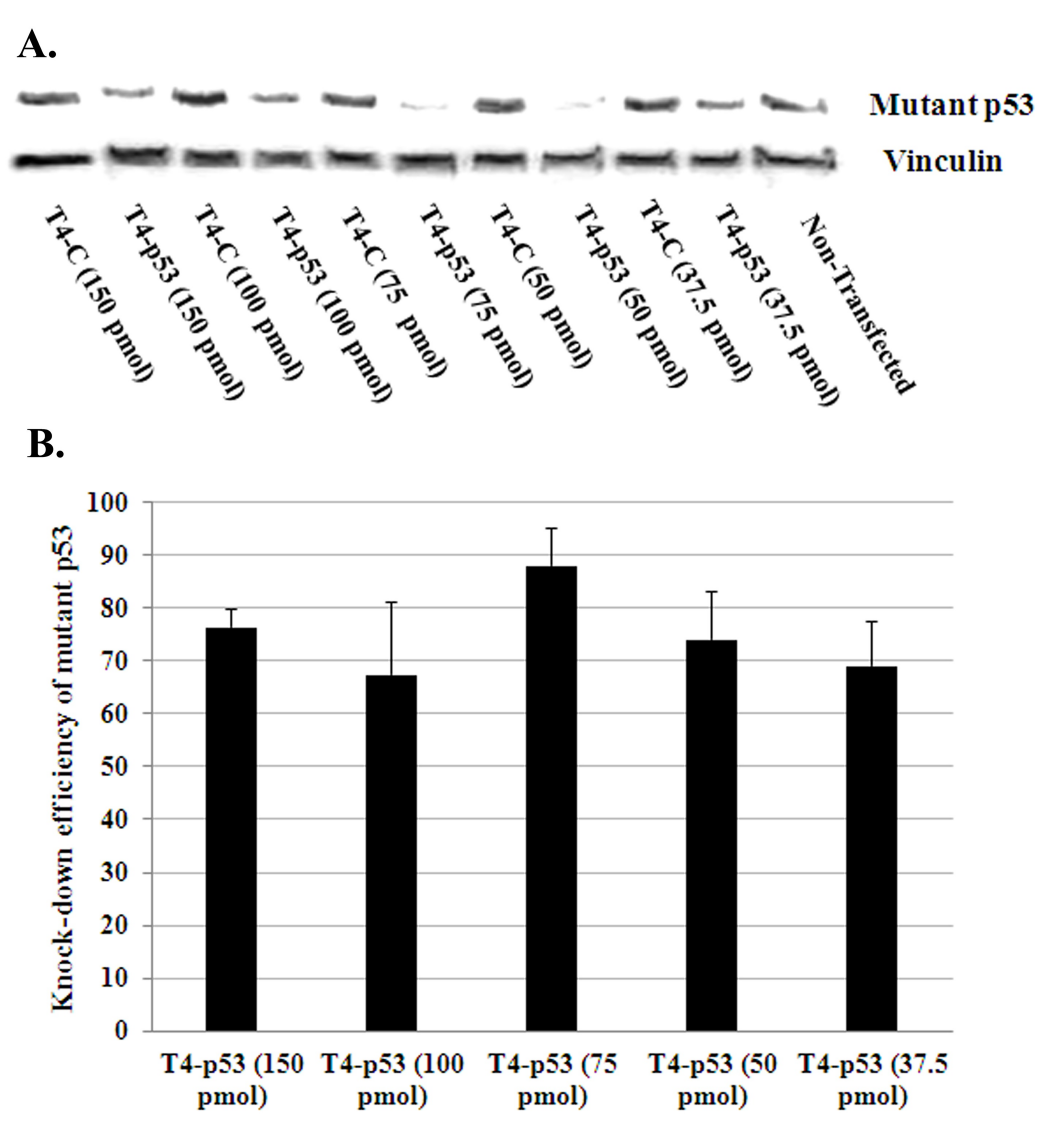
Determination of the optimal siRNA concentration to knockdown mutant p53. 318–1 cells were transfected with different concentrations of control or p53 siRNA encapsulated in T4 hybrid nanoparticles followed by western blotting for p53 and vinculin (Fig 4A). Graph represents summary of knockdown efficiency from 3 independent experiments (Fig 4B).

In order to determine the optimal siRNA concentration to knockdown mutant p53 (p53R172H) in 318–1 cells, T4 hybrid nanoparticles was used to encapsulate varying concentration of siRNA ranging from 37.5 nM to 150 nM. It was observed that T4 containing 75 nM siRNA was able to consistently produce the highest knockdown of mutant p53 (~88±3%) as shown by Western blot (Fig 4A). The transfection experiments and subsequent Western blotting to quantify the mutant p53 knockdown were repeated thrice and the results have been summarized in Fig 4B and Table 5.

**Table 5:** Knockdown efficiency of hybrid nanoparticle at different transfection media volume.

Nanoparticles	siRNA amount (μg)	siRNA conc. (nM)	Mutant p53 Knockdown efficiency (%)	
			Volume = 1 ml	Volume = 2 ml
T4	4.02	150	74±5	58±5
T4	2.68	100	69±6	62±4
T4	2.02	75	88±3	22±6
T4	1.34	50	75±4	1±3
T4	1.01	37.5	68±2	12±8

### 3.3. Effects of media volume, cell number and storage temperature on the knockdown efficiency of hybrid nanoparticles

The knockdown efficiency of the nanoparticles varies with the transfection media volume. Increasing the media volume from 1 ml to 2 ml caused a sharp decrease in the knockdown efficiency of T4 at different siRNA concentration (Table 5). For example, T4 entrapping 75 nM siRNA showed ~22±6% knockdown at 2 ml media volume vs. ~88±3% knockdown at 1 ml media volume. Similar pattern was also observed with T4 using different concentrations of siRNA (Table 5).

The Knockdown efficiency of the hybrid nanoparticles was affected by the storage temperature. This study was conducted up to 7 days of particle storage at 4°C and -20°C (Table 6). The cell viability was minimally affected by the nanoparticles stored at different temperature. The cell viability was recorded ~84±3% at Day1 *vs*. ~82±3% at Day7 when the particles were stored at 4°C, whereas, it was recorded ~67±2% at Day1 *vs*. ~72±4% at Day7 when they were stored at -20°C. On the contrary, the knockdown of mutant p53 was drastically reduced when the particles were stored at -20°C compare to 4°C. At -20°C, the silencing of mutant p53 was dropped to zero at Day7 from ~77±4% at Day1, whereas, at 4°C, the knockdown was reduced to ~37±4% at Day7 from ~60±5% at Day1 (Table 6).

**Table 6:** Knockdown efficiency of T4 stored at different temperature up to 7 days.

	Incubation Temp (4°C)		Incubation Temp (-20°C)	
Incubation period (Days)	Cell Viability (%)	Mutant p53 Inhibition (%)	Cell Viability (%)	Mutant p53 Inhibition (%)
1	84±3	60±5	67±2	77±4
2	82±2	55±10	66± 3	0
3	84±3	58±6	71±3	0
5	84±2	40±6	70±3	0
7	82±3	37±4	72±4	0

150 nM siRNA was encapsulated into T4.

The knockdown efficiency of these hybrid nanoparticles keeping siRNA concentration 150 nM was also monitored on different cell concentration (Table 7). The highest knockdown of mutant p53 (~73±3%) was achieved with a cell count of ~1.5x10^5^ and lowest (~26±7%) with a cell count of ~2x10^5^. However, increasing siRNA concentration from 150 nM to 350 nM didn’t improve the knockdown efficiency on a cell count of ~1.5x10^5^ (*e*.*g*. ~73±3% knockdown at 150 nM siRNA conc. *vs*. ~60±5% knockdown at 350 nM siRNA concentration) (Table 7).

**Table 7:** Mutant p53 knockdown efficiency of T4 using different amount of cells.

Nanoparticles	siRNA conc. (nM)	Cell count used in experiment	Cell viability (%)	Mutant p53 Inhibition (%)
T4	150	1x10^5^	68±5	57±6
T4	150	1.5x10^5^	85±4	73±3
T4	150	2x10^5^	89±2	26±7
T4	250	1.5x10^5^	83±4	60±4
T4	350	1.5x10^5^	77±3	60±5

### 3.4. Physicochemical characterization of hybrid nanoparticles—Particle size, morphology, and zeta potential

The particle size of the blank hybrid liposomes and siRNA-entrapped hybrid nanoparticles has been compared among different batches in the respective ranges of 30, 70 and 90 percentile (Table 3). PLGA and PLGA-PEG have shown differential impact on the size of the hybrid particles when they were used to gradually reduce lipid content in the formulations (Table 3). In case of hybrid siRNA-entrapped nanoparticles, the particle size was gradually reduced for T3, T4 and T5 when the lipid was replaced by PLGA compared to the respective blank liposomes (90 percentile). However, for T6, T7 and T8 where the lipid content was gradually replaced by PLGA-PEG, the particle size increased comparatively at 70 and 90 percentile with respect to the blank liposomes. Overall, the particle size at 70 percentile of T3 (~101±13), T4 (~100±8) and T5 (~125±11) was shown significantly smaller than T6 (~213±14), T7 (~217±4), and T8 (~216±8) when equal amount of lipid was replaced by either PLGA or PLGA-PEG in their respective formulations. On the other hand, when PLGA or PLGA-PEG was incorporated to replace lipid in the blank hybrid liposomes (F3-F8), the particle size was universally larger (at 30%, 70% and 90%) in those hybrid liposomes compared to blank liposomes (F1). The particle size of the blank liposomes (F1) at 90% was observed to be ~173nm, but when the lipid was reduced 5% (F3), 10% (F4) and 20% (F5) by PLGA, the particle size was observed ~256 nm, ~323 nm and ~328 nm, respectively. Whereas, when 5% (F6), 10% (F7) and 20% (F8) lipid was replaced by PLGA-PEG, the particle size of F6, F7 and F8 was noticed ~374 nm, ~364 nm and ~360 nm, respectively.

Cryo-transmission electron micrographs of only lipid (F1) and hybrid liposomes (F5 having PLGA and F8 having PLGA-PEG) are shown in Fig 2. Of the three hybrid liposomes from each group (F3, F4, F5 having PLGA and F6, F7, F8 having PLGA-PEG), the one with the lowest lipid and highest polymer content (*i*.*e*. F5 and F8) was chosen for imaging so as to understand the maximum changes in particle size brought by polymer incorporation. As shown in Fig 2, F5 formulation produced a comparable particle size to F1 but it was much smaller than F8.

The zeta potential of the blank hybrid liposomes and siRNA-entrapped hybrid nanoparticles was compared between PLGA- vs. PLGA-PEG-substitution (Table 4). PLGA-substituted hybrid liposomes had higher surface charge than that of PLGA-PEG-substituted hybrid liposomes. However, when the liposomes were used to entrap siRNA, both PLGA- and PLGA-PEG-substituted nanoparticles showed a comparable surface charge ranging from 41 to 46 mV.

### 3.5. Efficiency of siRNA encapsulation by hybrid nanoparticles

The efficiency of entrapment of siRNA by different hybrid nanoparticles (T1-T8) was determined by Ribogreen assay as shown in Fig 1. The siRNA encapsulation efficiency was gradually increased as lipid was substituted by polymer. For PLGA-substituted nanoparticles, the highest encapsulation efficiency (~65%) was reached at 10% PLGA (T4) and remained nearly unchanged at 20% PLGA (T5). On the other hand, a gradual increase of siRNA encapsulation was observed when the lipid was gradually reduced by PLGA-PEG. For example, the siRNA encapsulation efficiency was observed highest (~90%) by T8 (20% PLGA-PEG) compare to T6 (5% PLGA-PEG) which showed ~45% efficiency.

## 4. Discussion

The WTp53 gene is a tumor suppressor gene[33], and mutations of p53 gene are frequently detected in various cancers[34], resulting in drug resistance and hence poor prognosis. Advances in gene therapy in recent years have improved the outcome of cancer treatment. For example, the introduction of WTp53 in pancreatic adenocarcinoma showed a marked reduction in tumor volumes[35]. Another strategy is the use of RNAi technology to silence mutant p53[36]. The need of the hour is an appropriate delivery vehicle that could transport nucleic acids (*e*.*g*. DNA, RNA, siRNA) and other small molecule drugs into the tumor microenvironment.

Lipid nanoparticles developed previously in our lab succeeded in delivering siRNA to HCV-infected hepatocytes and brought about significant reduction in HCV replication[37, 38]. A modification of lipid-based nanoparticles containing high mobility group protein facilitated transfection of DNA into malaria parasite Plasmodium falcifarum-infected red blood cells without generating any cytotoxicity[39].

An improvement of lipid-based nanoparticles has been attempted in this study by partial substitution of lipid with FDA-approved highly tunable biocompatible synthetic organic polymer PLGA that mitigates cell cytotoxicity while enhancing knockdown efficiency. PLGA, a co-polymer of poly lactic acid (PLA) and poly glycolic acid (PGA), also has favorable degradation characteristics and helps in sustained release. Introduction of PEG moiety into PLGA has also been attempted so as to further reduce the hydrophobicity and negative charge on the surface and enhance interaction with the negatively charged siRNA. PLGA-PEG has been processed as diblock; PEG chains orient themselves towards the external aqueous phase in micelles, thus surrounding the encapsulated species. This improves the solubility of the nanoparticles and minimizes their aggregation[40]. Better release kinetics from diblock copolymers have been shown in comparison to PLGA alone[41].

siRNA technology has wide clinical applications now a days for post-translational gene silencing. The mechanism of action lies in binding to complementary mRNA of the targeted protein in a sequence-specific manner and causing its degradation. The major hurdle in siRNA transfection includes its poor intracellular uptake and enzymatic degradation *in vivo*. Polymers and lipids undergo electrostatic interactions with siRNA and form nanosized complexes which guard siRNA from degradation by nucleases, have better penetration due to its smaller size and facilitate cellular uptake of siRNA by endocytosis[37].

The present study is conducted with the formulation and optimization of lipid-polymer hybrid nanoparticles that could effectively deliver siRNA to silence mutant p53 in a mouse osteosarcoma cell line 318–1 with minimal cytotoxicity. Varying lipid/polymer ratio in these nanoparticles, we have tried to get the best combination possible while maintaining smaller particle size, high siRNA encapsulation, functional integrity of siRNA, and efficient mutant p53 knockdown. We have also tried to assess the effects of long-term storage of nanoparticles at different temperatures, as well as transfection media volume, on cell viability and p53 knockdown efficiency.

The nanoparticles developed earlier containing only lipid showed that the highest knockdown of mutant p53 is achievable with 150 nM siRNA. Therefore, the lipid-polymer hybrid nanoparticles were subsequently prepared incorporating 150 nM siRNA. The best nanoparticle in terms of cell viability and p53 knockdown efficiency from PLGA group (T3-T5) and PLGA-PEG group (T6-T8) was recognized as T4 and T6, respectively. T4 was advantageous over T6 in terms of its smaller particle size and cell viability and therefore was used to optimize the condition suitable for siRNA transfection. T4 containing 75 nM siRNA gave the highest knockdown of p53 as shown by Western blot. Therefore, the physicochemical properties of the nanoparticles were characterized at 75 nM siRNA rather than at 150 nM.

The knockdown efficiency of mutant p53 decreases significantly (~ 3 to 4 folds) with increasing the transfection media volume from 1 to 2 ml. Therefore, for all the experiments, the transfection media volume was maintained 1 ml. With long-term storage of the nanoparticles (7 days) at 4°C, the cell viability was maintained more than ~80%, while the p53 knockdown efficiency gradually decreased to a great extent. At -20°C, the cell viability reduced to ~67% and a drastic drop down in p53 knockdown efficiency was noticed just after one day. This could be due to the degradation of the siRNA or degradation of the lipid/siRNA complex with time and drastic temperature changes[38]. Under the given experimental conditions, the highest p53 silencing has been registered with a cell count of 1.5x10^5^/3 cm dish.

The recommendation for the best nanoparticle to be used among all the formulations that we generated has been done strictly based on the physical parameters of the nanoparticles as well as their effects in the biological systems. Considering the physicochemical parameters such as the particle size, zeta potential and siRNA encapsulation efficiency and effects on cell viability, the 10% PLGA-based nanoparticle T4 stands out to be the most suitable one. Transfection experiments on 318–1 cell line has shown that T4 with siRNA concentration of 75 nM gives the maximum knockdown of mutant p53 (nanoparticle:siRNA = 6.8:0.66) as observed from the Western blot analysis.

In conclusion, the nanoparticles designed in our lab are relatively easy to prepare, biodegradable, highly permeable, have low toxicity, and efficiently knockdown mutant p53 in 318–1 osteosarcoma cells. Though different types of nanoparticles are being administered for various clinical applications including viral diseases and cancers, their applications in the field of osteosarcoma are rare. This research is promising and may open some new avenues for the treatment of osteosarcoma. As such, this study warrants a future evaluation of this formulation for gene silencing efficiency of mutant p53 in animal models for the treatment of cancer.

## Supporting information

S1 Fig Identification of the formulation with the best knockdown efficiency by Western blotting. (TIF)

S2 Fig Determination of the optimal siRNA concentration to knockdown mutant p53. (TIF)

S1 Table Physiochemical characterization of different hybrid nanoparticles. (XLSX)
