# Supplementary figures and images for: Novel siRNA formulation to effectively knockdown mutant p53 in osteosarcoma
Source: PLoS One. 2017 Jun 21;12(6):e0179168. doi: 10.1371/journal.pone.0179168 (PMC5479560; doi:10.1371/journal.pone.0179168)

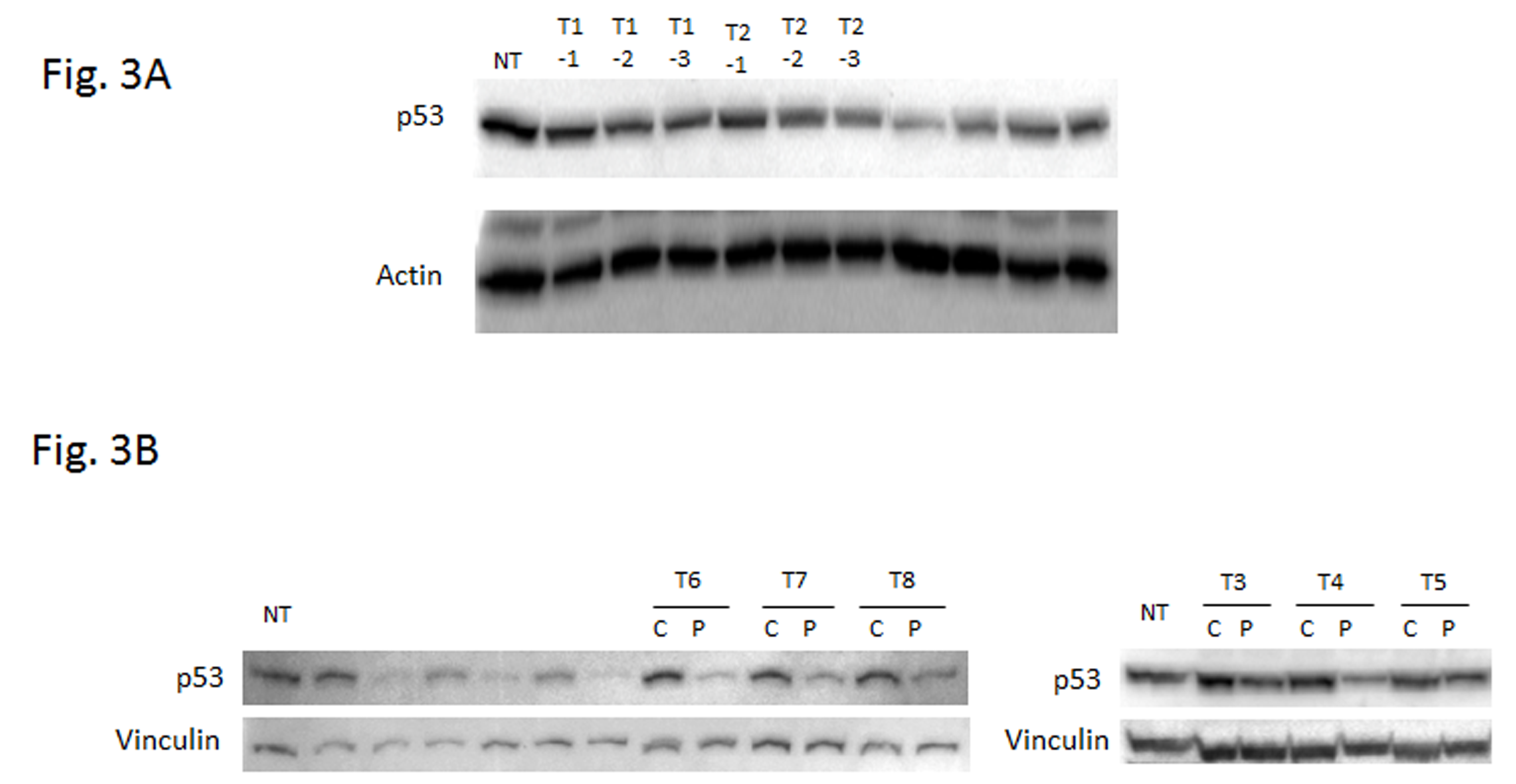

Supplement: S1 Fig — (TIF) [file pone.0179168.s001.tif]

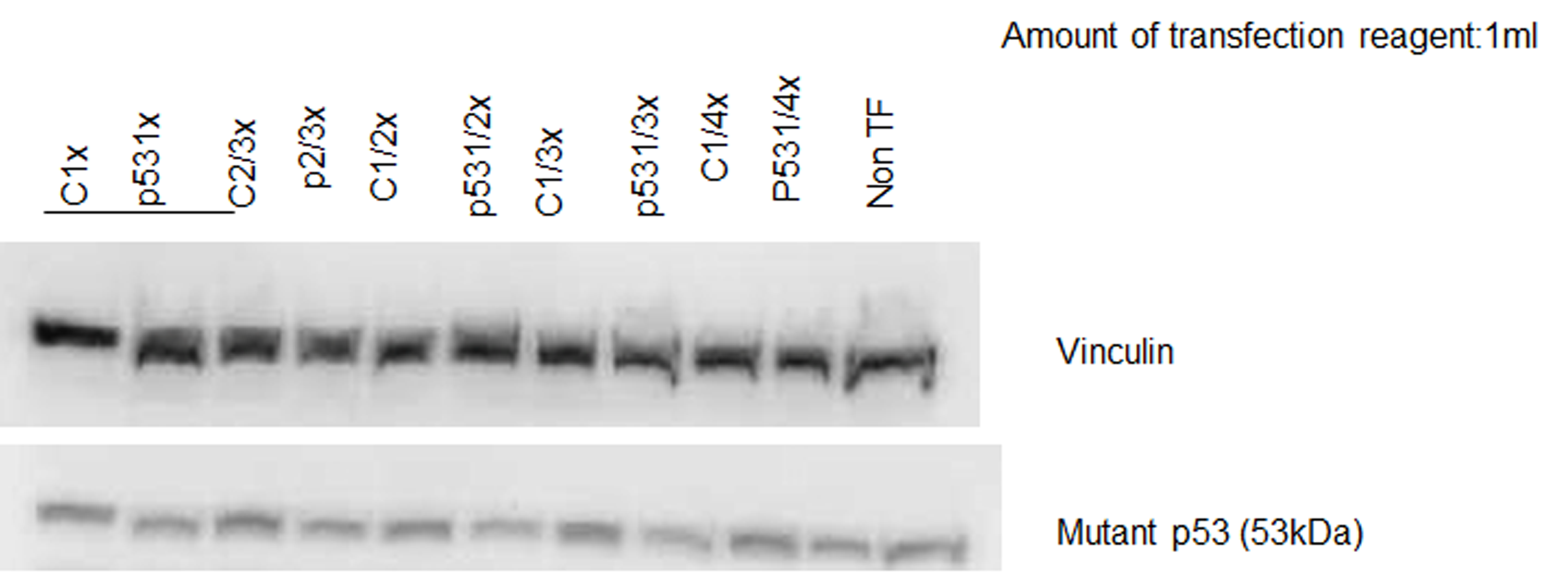

Supplement: S2 Fig — (TIF) [file pone.0179168.s002.tif]
